# Lessons Learned Serving Pregnant, Postpartum, and Parenting People with Substance Use Disorders in Massachusetts: The Moms Do Care Program

**DOI:** 10.1007/s10995-023-03775-5

**Published:** 2023-10-04

**Authors:** Laura Sternberger, Amy Sorensen-Alawad, Telyia Prescott, Hibiki Sakai, Kayla Brown, Norma Finkelstein, Amy Salomon, Davida M. Schiff

**Affiliations:** 1 Institute for Health and Recovery, Watertown, USA; 2 Massachusetts Department of Public Health, Boston, USA; 3 Advocates for Human Potential, Inc. Center for Research and Evaluation, Chicago, USA; 4 Advocates for Human Potential, Inc. Center for Research and Evaluation, Sudbury, USA; 5 Massachusetts General Hospital, Boston, USA

**Keywords:** Perinatal care, Substance use disorder, Peer recovery, Integrated care, Trauma-informed care

## Abstract

**Purpose:**

The purpose of this paper is to describe the design and implementation of a multidisciplinary, integrated approach to supporting pregnant, postpartum, and parenting people (PPPP) and their families affected by substance use disorders (SUD).

**Description:**

Between 2015 and 2022, the Moms Do Care (MDC) Program, sponsored by the Massachusetts Department of Public Health Bureau of Substance Addiction Services, established or expanded 11 co-located medical and behavioral health teams in locations across Massachusetts. These teams provided trauma-informed primary and obstetrical health care, SUD treatment and recovery services, parenting support, and case management for approximately 1048 PPPP with SUD.

**Assessment:**

By enhancing the capacity of medical and behavioral health providers offering integrated care across the perinatal health care continuum, MDC created a network of support for PPPP with SUD. Lessons learned include the need to continually invest in staff training to foster teambuilding and improve integrated service delivery, uplift the peer recovery coach role within the care team, improve engagement with and access to services for communities of color, and conduct evaluation and sustainability planning.

**Conclusion:**

MDC prioritizes trauma-informed integrated care, peer recovery, and commits to addressing inequities and stigma; thus, this program represents a promising approach to supporting PPPP impacted by SUD. The MDC model is relevant for those working to build multidisciplinary, integrated systems of health care and perinatal SUD services for marginalized populations.

## Significance

The Moms Do Care (MDC) Program is an integrated, multidisciplinary model of service provision originally created in response to the rising rates of perinatal opioid use disorder in Massachusetts. By partnering with and enhancing the capacities of medical, substance use disorder, and behavioral health providers along the perinatal continuum of care, and by leveraging the expertise of perinatal recovery coaches, MDC established and expanded webs of regional support for families impacted by any SUD. The MDC model is especially noteworthy to those building sustainable, multidisciplinary models of health care and perinatal SUD services for this population.

## Introduction

Massachusetts has been heavily impacted by the opioid epidemic. During the timeframe 2010–2020, there was a four-fold increase in the rate of opioid related overdoses (Massachusetts Department of Public Health, 2023). One in five pregnancy-associated deaths in Massachusetts were related to substance use between 2005 and 2014, increasing from 9% in 2005 to more than 40% in 2014 (Massachusetts Department of Public Health, 2018). The state has seen a significant rise in pregnancies affected by opioid use disorder and infants who experience symptoms of neonatal opioid withdrawal syndrome (Haight et al., 2018; Hirai et al., 2021). Despite known improvements in obstetrical and neonatal outcomes for pregnant people with a substance use disorder (SUD) cared for in multidisciplinary settings, very few programs provide co-located medical, SUD, and behavioral health services (Goler et al., 2008; Krans et al., 2018).

Moms Do Care (MDC) is an evolving, multidisciplinary approach to supporting pregnant, postpartum, and parenting people (PPPP) and their families impacted by SUD in Massachusetts. MDC was established in 2015 through a three-year, cooperative agreement from the Substance Abuse Mental Health Services Administration (SAMHSA), awarded to the Massachusetts Department of Public Health’s Bureau of Substance Addiction Services to provide low-barrier access to medication for opioid use disorder, opioid overdose prevention, and recovery support for pregnant people with a diagnosis of opioid use disorder in two underserved communities in the state. With additional state and federal resources, the Massachusetts Department of Public Health expanded the model to seven additional sites and broadened eligibility to include PPPP and their children impacted by SUD. This article summarizes the development, implementation, and expansion of the MDC model; describes program and participant characteristics; and discusses challenges, lessons learned, and areas to explore in more depth.

## Program Model

The MDC program goal is to meet the needs of the target population—PPPP with SUD—through integrated primary, obstetrical, pediatric, and behavioral health care services, which include SUD treatment, recovery coaching, parenting support, and case management.^1^

**Fig. 1:**
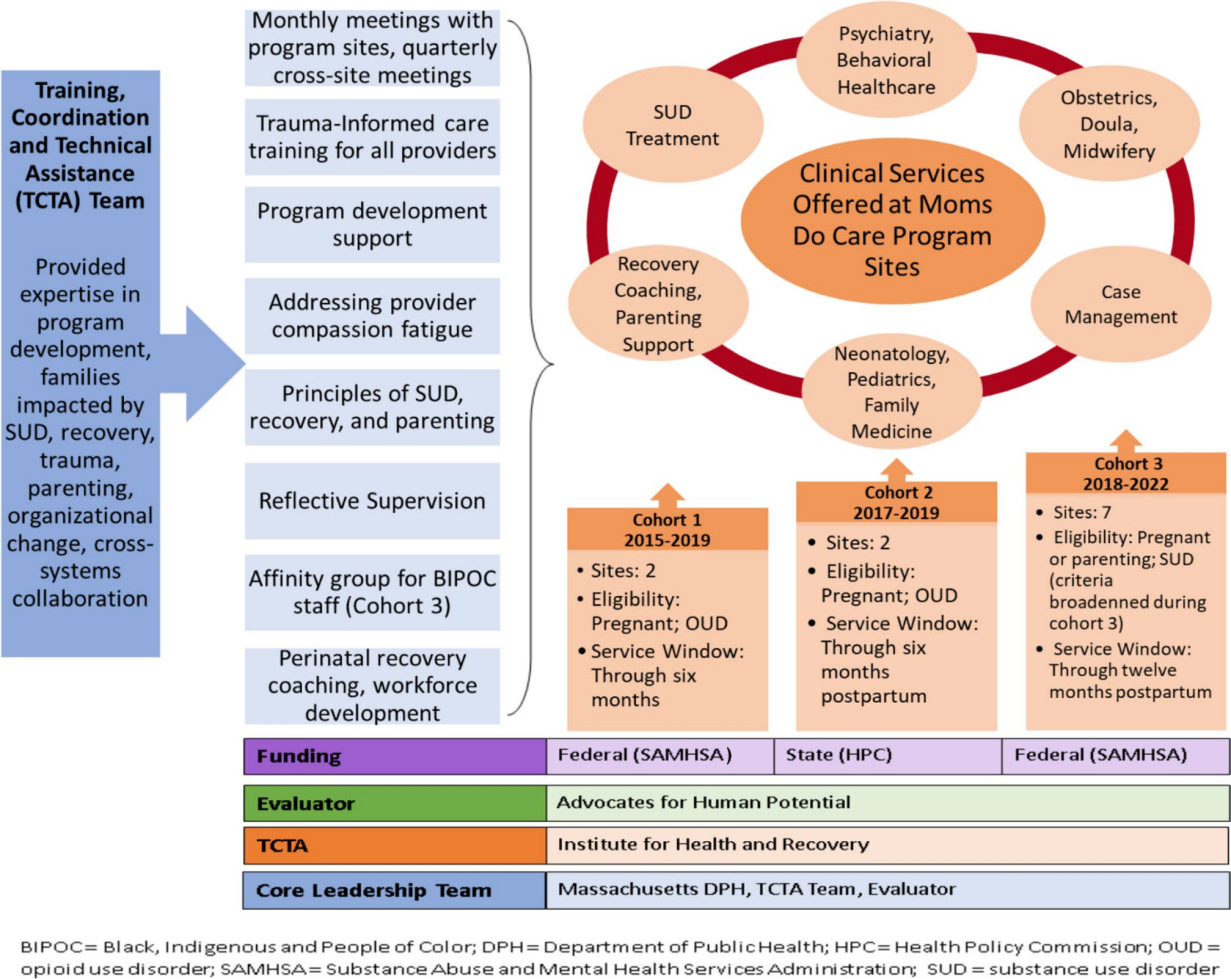
Components of the Massachusetts Moms Do Care Model (2015–2022)

The MDC program sites, located throughout the state (Institute for Health and Recovery, 2023), were selected through a competitive application process. Four birthing hospitals were selected as the initial MDC sites (Cohorts 1 and 2) to focus on opioid use disorder; they were funded from 2015 to 2019. In 2018–2022, the eligibility broadened, and MDC sites enrolled PPPP affected by a history of any SUD. (Fig. 1 illustrates the components of the MDC model.)

Table 1 describes each of the multidisciplinary site teams.

**Table 1:** Moms Do Care Program Model — Sites, Staffing, and Services

Moms Do Care site name	Medical providers	Behavioral health providers	Dedicated admin/data management staff	Perinatal recovery coach(es)	Medications for opioid use disorder (MOUD) prescriber(s) onsite, or off site with priority access	Staff size
**COHORT 1.** (Funded 2015–2019) **Eligibility:** Pregnant people meeting DSM-V criteria for opioid use disorder**; Service window:** through 6 months postpartum						
University of Massachusetts Memorial Medical Center (Central MA)	OB/GYN	Social work/therapy, psychiatry	Yes	Yes	Onsite	10 to 13
Cape Cod Hospital (Southeastern MA)	OB/GYN, addictions nursing	N/A	No	Yes	Off-site	10 to 15
**COHORT 2.** (Funded 2017–2019) **Eligibility:** Pregnant people meeting DSM-V criteria for opioid use disorder**; Service window:** through 6 months postpartum						
Beverly Hospital (Northeastern MA)	OB/GYN, midwifery, neonatology	Social work/therapy	No	Yes	Onsite	8 to 11
Lowell General Hospital^a^ (Northeastern MA)	OB/GYN, maternal and child health, pediatrics	Social work/therapy	Yes	Yes	Onsite	8 to 10
**COHORT 3. (**Funded 2018–2022) **Eligibility:** Pregnant *or* parenting people with a child 36 months or younger *and* any history of opioid use disorder (2018–2020), history of opioid use disorder and/or stimulant use disorder (2020–2021); any history of substance use disorder (2021–2022); **Service window:** through 12 months after enrollment						
Duffy Health Center^b^ (Southeastern MA)	Addiction Nursing, Primary Care	Social work/therapy, psychiatry	No	Yes	Onsite and Offsite	5 to 10
Massachusetts General Hospital (Boston/Metrowest)	OB/GYN, pediatrics, midwifery, family medicine	Social work/therapy, psychiatry	Yes	Yes	Onsite	10 to 15
Whittier Street Community Health Center (Boston/Metrowest)	N/A	Social work/therapy, psychiatry, outreach worker	No	Yes	Onsite	3 to 6
Brockton Neighborhood Health Center (Southeastern MA)	OB/GYN, addictions nursing, midwifery, primary care, dietetics	Social work/therapy, psychiatry	No	Yes	Onsite and Offsite	8 to 12
Lynn Community Health Center (Northeastern MA)	OB/GYN, addictions nursing, family medicine	Social work/therapy, psychiatry, psychology, community health worker	No	Yes	Onsite	18 to 20
Lahey–Beverly Hospital (Northeastern MA)	OB/GYN, midwifery, neonatology	Social work/therapy	Yes	Yes	Onsite	8 to 11
Baystate-Franklin Medical Center (Western MA)	OB/GYN, addictions nursing, pediatrics, midwifery, ^c^Doula	Social work/therapy	Yes	Yes^c^	Onsite	12 to 15

^a^The Lowell General Hospital MDC Program was sustained through a partnership with a community health center, so it was only funded 2017–2019 under the mechanisms described in this article

^b^With the onset of new funding in 2018, the Cape Cod MDC program transitioned from Cape Cod Hospital to Duffy Health Center

^c^Baystate-Franklin Medical Center employed a unique model that utilized peer recovery coaches with standard training and peer recovery coaches trained as doulas

### Program Leadership and Staffing

The MDC core leadership team was comprised of representatives from the Massachusetts Department of Public Health; the training, coordination, and technical assistance (TCTA) vendor^2^; and an external program evaluator.^3^ The leadership team oversaw program development, implementation, and evaluation. Site provider staff met regularly with the TCTA team to address challenges and share lessons learned across the state.

### Participant Demographics and Clinical Characteristics

Data gathered between 2015 and 2021 shows that MDC participants across the three cohorts had complex social, economic, and behavioral health needs (Table 2).

**Table 2:** Self-reported demographic and clinical characteristics of MDC population at baseline

Characteristic (% or mean)	Cohort 1	Cohort 2	Cohort 3	All cohorts
	*n* = 187	*n* = 112	*n* = 489	*n* = 788
Age in years	28	30	31	29
White only	73.3	86.7	86.1	83.1
Black/African American only	1.6	0.0	4.1	2.9
More than one race	18.2	8.9	8.4	10.8
Hispanic/Latino	8.6	12.4	12.2	11.4
Less than high school	16.6	17.7	19.4	18.5
Unemployed	74.3	67.3	69.9	70.6
Monthly income	$691.67	$1,187.59	$767.15	$882.13
Unstably housed	59.9	74.3	54.4	58.6
Percentage of people currently pregnant	100.0	92.9	49.1	67.4
Percentage of families with at least 1 child	65.2	57.5	83.4	75.4
Of those with children, % with a child in protective custody	47.5	43.1	41.2	42.9
Used drugs at least weekly past year	82.4	74.3	50.9	61.7
Ever overdosed in lifetime	56.2	48.7	50.9	51.8
Physical abuse in lifetime	82.1	69.2	74.2	75.2
Sexual molestation/rape in lifetime	69.0	58.5	59.5	62.3
1+ traumatic events as minor	80.2	71.7	69.7	73.9
Depression in lifetime	82.4	73.5	71.4	74.3
Anxiety in lifetime	89.3	83.2	85.1	85.8
Suicide attempts in lifetime	26.7	27.4	25.6	26.1
Meets clinical PTSD criteria	45.5	31.0	31.1	34.5
Multiple problems count^b^	4.2	3.9	3.10	3.7

^a^Evaluation data presented in Table 2 were collected through October 3, 2021 (N = 788). This represents a subset of the total program population enrolled during the 2022 funding cycle ending September 30, 2022 (N = 1048). We focus on this subset because they were administered the most comprehensive instrument over the study time period. The full MDC program utilized the Government Performance Results Act (GPRA) data collection tool as required by the funder. In addition, through October 3, 2021, we incorporated several more components into patient interviews to assess domains of interest including opioid overdose history, trauma history and symptoms, frequency of drug use, mental health history, and addiction recovery measures

^b^Evaluators created a measure of eight specific problems that could potentially impact successful recovery and drive program service needs. The “problem count” was created from a series of GPRA, local questions, and standard scales including unemployment (GPRA); low educational attainment (GPRA), unstable housing (GPRA), negative social consequences of substance use (GPRA); substance use initiated before age 12 (local); traumatic abuse in childhood (local); PTSD at clinical levels, PTSD Checklist—Civilian Version (Weathers et al., 1994); and severity of mental health problem, Addiction Severity Index Psychiatric Composite Score above national norm for women in treatment programs, (McLellan et al., 2006)

As documented by the evaluation, between 2015 and 2021,^4^ MDC evaluated 788 PPPP with SUD. (This is a subset of the 1048 participants served between 2015 and 2022). Of those evaluated, 83% identified as white only, 3% as Black/African American only, and 11% as more than one race. In addition, 11% also identified their ethnicity as Hispanic. The mean age at enrollment was 29 years. All participants in Cohort One and 93% in Cohort Two were pregnant when they entered the MDC program. (A waiver process was initiated with Cohort Two that allowed, in rare instances and with approval, women who were no longer pregnant to enroll if they had just delivered or miscarried at the time of enrollment.) Due to the expanded eligibility criteria in Cohort Three (which allowed any postpartum people to enter the program), 49% of participants were pregnant upon entering the MDC program. (Refer again to Table 1). Seventy-five percent of all participants enrolled had at least one child. Seventy-one percent of participants were unemployed at enrollment in the MDC program and had monthly incomes below the federal poverty level ($1,919 for a family of 3) and 18% had not completed high school. Fifty-nine percent of participants were unstably housed. Among participants with children at enrollment (N = 594 across the three cohorts), 43% had at least one child currently involved in the child welfare system. Sixty-two percent of MDC participants self-reported using drugs weekly during the past year at the time of their enrollment, and 52% reported experiencing at least one prior overdose. Seventy-five percent reported a history of a traumatic event as a minor, including physical and/or sexual abuse. Rates of self-reported anxiety (86%) and depression (74%) were also high, and 26% had made a previous suicide attempt.

## Challenges and Lessons Learned in MDC Program Implementation

Several challenges and lessons learned emerged from reviewing meeting minutes and onsite trainings related to the implementation of this intertwining model of direct service, capacity building, and sustainability initiatives. The MDC site providers and TCTA team collaborated in developing strategies to address challenges and share lessons learned across the state.

### Cross-System Trauma-Informed Care Trainings Enhanced Collaboration and Care Integration

MDC faced challenges integrating two unique but complementary systems of care delivery: traditional medical care and SUD care for pregnant, postpartum, and parenting people. To meet this challenge, the MDC site providers and TCTA leadership team identified professional development opportunities to promote cross-system relationship building. Universal staff trainings were conducted on the principles of trauma-informed care (i.e., safety, trustworthiness, transparency, self-reflection, peer support, collaboration, empowerment, and cultural humility) (Fleishman et al., 2019). The MDC TCTA team also provided extensive individualized, site-based support for designing trauma-informed teams and leadership. Cross-team training addressed ways to build recovery-oriented systems of care. These trauma-informed, recovery-oriented care trainings and technical assistance created opportunities to improve care practices as well as enhance multidisciplinary, cross-system provider relationships and collaboration. The training and individualized technical assistance offerings were available to all MDC site staff and any regional providers serving this population. MDC site staff also received training in reflective supervision, a time for supervisees to examine their reactions to working with families from marginalized communities and inform the services they provide (Schmelzer & Eidson, 2020).

### Uplifting the Essential Role and Expertise of Perinatal Peer Recovery Coaches

Peer recovery coaching has been identified as a promising approach for improving engagement and retention of participants in SUD treatment programs (Bassuk et al., 2016). However, less is known about the impact of perinatal peer recovery coaches working with PPPP in integrated health care settings (Stowell et al., 2022). Although creating nonhierarchical, multidisciplinary teams that incorporate and leverage lived experience is challenging, perinatal peer recovery coaches who specialize in working with the PPPP population were essential for MDC care teams. Perinatal peer recovery coaches were critical to all aspects of program implementation, including participant recruitment and retention, training and capacity building, and medical culture change. Perinatal peer recovery coaches served as role models to both participants and providers as they administered screening and intake tools, provided recovery coaching, parenting support, case management, and advocacy. Moreover, perinatal peer recovery coaches modeled what wellness in recovery looks like. Some perinatal peer recovery coaches served as recovery experts in organizational change initiatives and statewide policy development.

While the role of a perinatal peer recovery coach was central to every facet of the MDC model, hiring and retaining perinatal peer recovery coaches, particularly those who identify as Black, Indigenous, and People of Color (BIPOC) had its challenges. Peer recovery coaches who support PPPP are highly specialized. People in recovery who sought employment as perinatal peer recovery coaches often had minimal professional health care experience; thus, ongoing orientation, recovery coach training, professional development, and supervisory support had to be provided. Research has shown that low salaries, inflexible human resource policies, the complicated life circumstances of people who are parenting in recovery, the intense and sometimes “triggering” nature of the work, and general health care staffing shortages affect the hiring and retention of peer recovery coaches (Costa & Friese, 2022).

Because perinatal peer recovery coaches positions required lived experience in SUD recovery and parenting, MDC sites had to engage their human resources departments to address the barriers people in recovery often face when entering the workforce. For example, they needed to advocate for flexibility in human resources policies for individuals with adverse driving records and prior criminal justice involvement. Human resources departments were encouraged to view lived experience in SUD recovery as relevant work experience—thereby providing justification for offering these individuals more competitive wages. Other factors important for recruiting and retaining a perinatal peer recovery workforce included implementing flexible work schedules, addressing the pervasive influences of racism and stigma related to people who have SUD, and providing guidance to MDC site and regional providers in understanding the role of perinatal peer recovery coaches and how they can have a wide-reaching impact in health care settings. To address the shortage of perinatal peer recovery coaches in the workforce, MDC developed a mentoring program for PPPP with a history of SUD. Some were past MDC participants. Furthermore, having supervisors with knowledge and expertise about SUD recovery was key to retaining perinatal peer recovery coaches. Most perinatal peer recovery coach supervisors were medical or behavioral health professionals practicing at MDC sites who could advise on clinical, child welfare, and case management concerns; they were also trained in the principles of recovery coaching.

### Expanding Access to Communities of Color

Federal funding designated specifically to address the opioid crisis was used to implement the MDC program. In Massachusetts, most people with opioid use disorder identify as white (Massachusetts Department of Public Health, 2023), resulting in a disproportionately white participant population in the MDC program. As MDC evolved and program data became available, it became clear that the program needed to focus on identifying and meeting the needs of diverse, minority populations in the state who were not eligible for MDC services. The MDC sites selected for Cohort 3 included several Black- and Latinx-serving community health centers, providing opportunities to engage more diverse populations. Also, in 2020, with new federal funding to support the program (that lessened restrictions on eligibility criteria), MDC could offer services to PPPP with any type of SUD, not just those with only an opioid use disorder. This enhanced the program’s ability to enroll individuals with SUD who identified as BIPOC, and increased understanding of how the model should be adapted to serve these communities.

MDC also worked to engage with BIPOC staff more intentionally. In 2020, in response to the heightened stress associated with the COVID-19 pandemic and the nationwide focus on racial injustice, the TCTA team initiated an affinity group for MDC staff identifying as BIPOC. The group was facilitated by a BIPOC TCTA staff member. It was hosted during paid work hours and offered BIPOC staff who work in predominantly white spaces a reparatory time to gather weekly in solidarity for supportive, reflective conversation and to provide staff a non-othering space through racial and cultural affinity to share their experiences.

### Creating Nonjudgmental and Coordinated Webs of Regional Support

Establishing trust is time consuming yet fundamental to participant engagement and retention. Research demonstrates that generations of mistrust, systemic trauma, and institutional disengagement may be a protective response to the persistence of structural racism and endemic mistreatment (Polanco-Roman et al., 2016). PPPP impacted by SUDs face stigma, shame, and fears related to loss of child custody. Potential participants often required weeks, if not months, of engaging with MDC staff before they felt trustful enough to enroll in the program.

MDC staff established and strengthened regional provider collaborative groups to help remove referral barriers and identify other effective outreach and engagement strategies. These groups aimed to create a nonjudgmental and coordinated web of community-based services along the prenatal to early childhood continuum served by the MDC program. Individuals who met eligibility criteria could enter care at any point in the continuum. They could sign a release of information, enabling cross-system collaborators to communicate. To facilitate creating a nonjudgmental and “no wrong door” approach to accessing care, the MDC TCTA team used these regional provider groups to offer training in topics that included motivational interviewing and trauma-informed, recovery-oriented, and harm reduction practices specific to this population (Pregnancy and Substance Use: A Harm Reduction Toolkit—National Harm Reduction Coalition, 2023).

### Evaluating and Sustaining MDC

MDC had a robust evaluation component. A lengthy intake process and follow-up data collection tools were critical to understanding the participant population and informing quality improvement and sustainability. However, the intensive data collection at the onset of service provision was intrusive and disconnected from the larger goal of providing care to an already distrustful and historically traumatized group. Furthermore, the evaluation design was challenged by the evolving nature of the model and resulting shifts in staffing; changes to participant eligibility criteria; and modifications to data collection requirements, tools, and guidance. To reduce the potentially intrusive nature of data collection, MDC sites used program staff rather than professional evaluators to conduct the baseline interview as part of program intake. This enabled the team to build trust and engage with participants while also assessing their readiness for the interview. Future manuscripts will describe the data collection and analysis strategies, service utilization, and maternal outcomes needed to understand the program’s effectiveness.

To promote sustainability, the MDC team advised sites to advocate to clinic and hospital leadership for support of critical, yet nonbillable, services such as data collection, care coordination, cross-agency collaboration, and capacity building to sustain the provision of services after federal funding ends. The team also convened with state insurance payors to discuss opportunities to sustain the MDC model after grant funding ends. Discussions on reimbursement for MDC’s integrated, multidisciplinary services are ongoing, as are conversations with the state Medicaid agency to create a PPPP bundled reimbursement structure that addresses the cost and productivity considerations of coordination and collaboration between multiple system partners (i.e., state child welfare and public health departments, health care organizations, and community agencies).

## Conclusion

The Moms Do Care program developed an evidence-informed model of care targeted specifically at PPPP with SUD. MDC’s core components are continually evolving to include co-located perinatal, primary, pediatric, and behavioral health care; SUD treatment; case management; and peer recovery support. Training and technical assistance activities focused on improving direct care services for this population, as well as creating a recovery-oriented, trauma-informed environment that benefits participants, providers, and the systems they work in. Current priorities include addressing the impact of structural racism on service access, because participants in MDC remain overwhelmingly white, despite concerted efforts to serve communities of color. Finally, health care organizations that intend to utilize specialized peer recovery coaches as part the perinatal service provision team must be prepared to invest time and financial resources to train all providers in the continuum of care about trauma-informed, recovery-oriented approaches and shift traditional organizational and hiring cultures to be more inclusive of people with a history of SUD. Future research and evaluation on this population is needed to better understand the impact of programs for PPPP with SUD, and the efficacy of perinatal peer recovery coaches.

## Data Availability

Data collected for the purposes of this manuscript is owned by the Massachusetts Department of Public Health. MDPH is available for additional inquiry into its use.

## Footnotes

1 The MDC program design and evaluation were conducted in accordance with prevailing ethical principles. The program was approved by the Massachusetts Department of Public Health Institutional Review Board.

2 Institute for Health and Recovery: https://www.healthrecovery.org/

3 Advocates for Human Potential: https://www.ahpnet.com/

4 Evaluation data presented in this section and Table 2 were collected through October 3, 2021 (N = 788). This represents a subset of the total program population enrolled during the 2022 funding cycle ending September 30, 2022 (N = 1048). We focus on this subset because they were administered the most comprehensive instrument over the study time period, including the funder’s required Government Performance and Results Act (GPRA) measure as well as locally selected standard scales and question sets to assess domains outlined here. These include opioid overdose history (local questions); substance use disorder history and frequency (Global Assessment of Individual Needs, Short Screener from Dennis et al., 2006); trauma history (local questions); post-traumatic stress disorder (PTSD) symptoms (PTSD Checklist Civilian Version from Weathers et al., 1994); and lifetime rates of mental health conditions (modified GPRA). After October3, 2021, the evaluation collected GPRA data only.
